# METTL14/IGF2BP1 m^6^A axis promotes pyroptosis in *Streptococcus pneumoniae*-induced pneumonia by regulating NEK7 mRNA stability

**DOI:** 10.1128/iai.00474-25

**Published:** 2026-02-12

**Authors:** Cheng Chen, Di Zhang

**Affiliations:** 1 Respiratory and Critical Care Medicine Department, Caidian District People’s Hospital, Wuhan, China; 2 Infectious Disease Department, Caidian District People’s Hospital, Wuhan, China; University of Illinois Chicago, Chicago, Illinois, USA

**Keywords:** *Streptococcus pneumoniae*-induced pneumonia, m^6^A, METTL14, pyroptosis, NEK7, IGF2BP1

## Abstract

*Streptococcus pneumoniae* (*S. pneumoniae*) infection induces pyroptosis in human pulmonary artery epithelial cells (HPAEpiCs), which contributes to pneumonia pathogenesis. We aimed to investigate the regulatory role of N6-methyladenosine (m^6^A) modification mediated by methyltransferase-like (METTL) 14 in this process and elucidate the underlying molecular mechanisms. HPAEpiCs were infected with *S. pneumoniae*. Cell viability was assessed using the cell counting kit-8 assay, while cytokine concentrations were measured by enzyme-linked immunosorbent assay. Pyroptosis levels were analyzed through flow cytometry and Western blot for pyroptotic protein expression. Gene expression profiles, protein-RNA interactions, and m^6^A methylation sites were characterized by quantitative reverse transcription-polymerase chain reaction, RNA immunoprecipitation, and dual-luciferase reporter assays. *In vivo* experiments involved intranasal administration of *S. pneumoniae* in mice to evaluate pulmonary pathological changes. *S. pneumoniae* D39*-*infected HPAEpiCs exhibited enhanced pyroptosis and adhesive/invasive capabilities, accompanied by elevated m^6^A modification mediated by METTL14. In addition, METTL14 inhibition suppressed pyroptosis and adhesive/invasive capabilities and ameliorated *S. pneumoniae* D39*-*induced lung injury. Notably, NEK7 overexpression reversed the pyroptosis reduction caused by METTL14 knockout. Mechanistically, the METTL14/insulin-like growth factor 2 mRNA-binding proteins (IGF2BP)1 m^6^A regulatory axis modulated NEK7 mRNA stability through m^6^A-dependent post-transcriptional regulation. The METTL14/IGF2BP1 m^6^A regulatory axis promoted *S. pneumoniae* D39*-*induced pyroptosis by stabilizing NEK7 mRNA transcripts. Targeting this m^6^A regulatory pathway represents a potential therapeutic strategy for managing *S. pneumoniae* D39*-*induced pneumonia.

## INTRODUCTION

Bacterial pneumonia is a severe respiratory infection that poses a significant threat to public health (1). Among various pathogens, *Streptococcus pneumoniae* (*S. pneumoniae*) is a leading cause of pneumonia (1). This gram-positive pathogen colonizes the upper respiratory tract and invades lung tissues, triggering severe inflammation and alveolar damage (2). Although *S. pneumoniae* is traditionally regarded as an extracellular pathogen, growing evidence suggests it can adhere to and invade respiratory epithelial cells, establishing intracellular niches. Early studies using lung tissue biopsies and murine infection models confirmed that *S. pneumoniae* can invade both alveolar epithelial cells and cerebral vascular endothelial cells, forming intracellular infection foci (3, 4). Subsequent research has shown that this invasion is mediated by bacterial surface adhesins—such as phosphocholine, pneumococcal surface protein A, and choline-binding protein A—which interact with host receptors like the platelet-activating factor receptor to promote clathrin-dependent endocytosis (5). High-risk populations include young children, the elderly, and immunocompromised individuals, with an estimated 1.6 million annual deaths attributed to pneumococcal diseases (6). *S. pneumoniae* induces pneumonia through a series of pathological mechanisms, including adherence to respiratory epithelial cells, invasion of underlying tissues, and activation of the host’s immune response—which leads to excessive inflammation and cytokine release—and subsequent damage to lung parenchyma and impairment of gas exchange (7). Current treatments rely on antibiotics (e.g., β-lactams, macrolides) and polysaccharide vaccines; however, rising antibiotic resistance (e.g., penicillin-nonsusceptible strains) and limited vaccine serotype coverage underscore unmet clinical needs (7), highlighting the urgent demand for novel therapeutic strategies.

Pyroptosis, a highly inflammatory form of programmed cell death, plays a central role in the host response to *S. pneumoniae* infection (8). It is initiated by inflammasome activation. These multiprotein complexes recognize pathogen-associated molecular patterns, triggering the cleavage of pro-caspase-1 (pro-CASP1) into its active form (CL-CASP1) and the secretion of pro-inflammatory cytokines (8). Pro-CASP1, the zymogen form of caspase-1, remains inactive in healthy cells. Pathogen exposure or inflammatory stimuli activate signaling pathways that drive the assembly and activation of inflammasomes, such as the nucleotide-binding oligomerization domain-like receptor family pyrin domain-containing 3 (NLRP3) inflammasome, which recruits pro-CASP1 for auto-cleavage into CL-CASP1 (9). In the context of NLRP3 inflammasome activation, CL-CASP1 contributes to pyroptosis by cleaving gasdermin D (GSDMD). The N-terminal fragment of GSDMD then oligomerizes, inserts into the plasma membrane, forms pores, and leads to cell lysis (9). Additionally, CL-CASP1 processes pro-cytokines such as interleukin (IL)-1β and IL-18, converting them into their mature, bioactive forms for secretion and subsequent amplification of the inflammatory response (10). A deeper understanding of pyroptosis mechanisms in *S. pneumoniae* D39*-*induced pneumonia is crucial for developing targeted therapeutic interventions.

N⁶-methyladenosine (m⁶A) is the most prevalent internal modification in eukaryotic messenger RNAs (11). This dynamic and reversible epitranscriptomic mark is deposited by methyltransferases (“writers”), removed by demethylases (“erasers”), and specifically recognized by m⁶A-binding proteins (“readers”) (11). Core components of this regulatory system include the writer complex methyltransferase-like (METTL)3–METTL14, erasers such as fat mass and obesity-associated protein (FTO) and AlkB homolog 5 (ALKBH5), and reader proteins—particularly the YTH domain-containing family (YTHDFs)—which selectively bind m⁶A-modified RNAs. The functional roles of m⁶A modification in lung-related diseases are increasingly being elucidated. For example, Wu et al. (12) demonstrated that histone lactylation-regulated METTL3 promotes ferroptosis via m^6^A modification of acyl-CoA synthetase long-chain family member 4 (ACSL4) in sepsis-associated lung injury. Moreover, Yang et al. (13) revealed that knockdown of METTL14 suppresses the malignant progression of non-small cell lung cancer by reducing Twist expression. However, most studies exploring m^6^A modification in the context of pneumonia have primarily focused on viral pneumonia, such as coronavirus disease 2019 (14). Notably, limited research has addressed its role in bacterial pneumonia.

NIMA-related kinase 7 (NEK7) is a member of the NIMA-related kinase family and has been identified as a critical regulator of NOD-like receptor family pyrin domain-containing 3 (NLRP3) inflammasome activation, which is closely linked to pyroptosis (15). Recent studies demonstrate that NEK7 interacts with NLRP3 to facilitate the assembly and activation of the inflammasome complex, ultimately triggering caspase-1 activation and subsequent pyroptosis (16). However, the upstream regulatory mechanisms governing NEK7 function in the context of *S. pneumoniae* D39*-*induced pneumonia, particularly its potential modulation by m^6^A RNA modification, remain poorly understood.

This study aimed to investigate the regulatory role of METTL14-mediated m^6^A modification in pyroptosis during *S. pneumoniae-*induced pneumonia and to elucidate the underlying molecular mechanisms. By exploring the interplay between m^6^A modification, NEK7, and pyroptosis, we seek to provide novel insights into the pathogenic mechanisms of *S. pneumoniae* D39*-*induced pneumonia.

## MATERIALS AND METHODS

### Cell culture, treatment, and transfection

Human pulmonary alveolar epithelial cells (HPAEpiCs; #3200; Zhong Qiao Xin Zhou Biotech Co., Ltd., Shanghai, China) were maintained in their manufacturer-recommended medium (#3201; Zhong Qiao Xin Zhou Biotech) supplemented with 10% heat-inactivated fetal bovine serum, 1% penicillin/streptomycin, and 1% epithelial cell growth supplement at 37°C in a 5% CO₂ incubator.

For *S. pneumoniae* treatment (serotypes D39 and E1), pneumococcal strains D39 (serotype 2) and E1 (serotype 3) were cultured in Todd-Hewitt broth supplemented with 0.5% yeast extract (THY) to the mid-logarithmic phase (OD₆₀₀ ≈ 0.4–0.5). The bacteria were then washed twice and resuspended in phosphate-buffered saline (PBS). HPAEpiCs were seeded in six-well plates and allowed to reach approximately 80% confluence. Prior to infection, the cell culture medium was replaced with antibiotic-free medium. The cells were infected with *S. pneumoniae* resuspended in PBS (1 × 10⁸ CFU/mL) at a multiplicity of infection of 10, while the control group was treated with an equal volume of inactivated *S. pneumoniae* induced by heat. After a 2-h co-incubation period at 37°C in a 5% CO₂ incubator, the cells were washed three times with PBS to remove non-adherent bacteria. To kill any remaining extracellular bacteria, the cells were then incubated in fresh medium containing gentamicin (20 μg/mL) and penicillin G (0.5 μg/mL) for an additional 22 h at 37°C in a 5% CO₂ incubator. In addition, HPAEpiCs were treated with Z-VAD-FMK (#S7023; purity: 99.79%; Selleck Chemicals, Houston, TX, USA), VXX-765 (#HY-13205; purity: 99.99%; MedChem Express, Monmouth Junction, NJ, USA), Ferrostatin-1 (#SML0583; purity: ≥95%; Sigma-Aldrich, St. Louis, MO, USA), 3-MA (#HY-19312; purity: 99.91%; MedChem Express), and Necrostatin-1 (#HY-15760; purity: 99.89%; MedChem Express) for 24 h.

For transfection, when HPAEpiCs reached 80% confluence, short hairpin RNA targeting METTL14 (shMETTL14#1 and #2) and insulin-like growth factor 2 mRNA-binding protein 1 (IGF2BP1) (shIGF2BP1) as well as NEK7 overexpression plasmids and the negative controls (shNC and empty vector) were transfected using a commercial transfection reagent (#L3000001; Thermo Fisher Scientific, Waltham, MA, USA) according to the manufacturer’s instructions. After 6 h, the transfection medium was replaced with fresh complete medium, and cells were cultured for an additional 48 h at 37°C in a 5% CO₂ incubator for subsequent analyses.

### Cell counting kit-8 assay

HPAEpiCs were seeded in 96-well plates at a density of 5 × 10^3^ cells per well. After adherence, the cells were incubated for 0, 4, 8, 12, and 24 h. At each time point, 10 μL of cell counting kit-8 (CCK-8) solution (#BS350A; Biosharp Biotech Co., Ltd., Hefei, China) was added, and the cells were further incubated for 2 h. Absorbance at 450 nm was measured using a microplate reader. Cell viability was calculated as a relative percentage (%) of the 0-h value.

### Enzyme-linked immunosorbent assay

An enzyme-linked immunosorbent assay (ELISA) experiment was conducted to quantify the concentrations of IL-18 and IL-1β in HPAEpiCs at 0, 4, 8, 12, and 24 h post-incubation. Cytokine levels in cell supernatants were measured using commercial ELISA kits (IL-18: #ml058055 and IL-1β: #ml058059; Enzyme-Linked Biotechnology Co., Ltd., Shanghai, China) according to the manufacturer’s instructions. Absorbance at 450 nm was measured using a microplate reader, and cytokine concentrations were calculated from the standard curves provided by the manufacturer.

### Flow cytometry

At 0, 4, 8, 12, and 24 h post-incubation, both adherent and non-adherent HPAEpiCs were analyzed to account for potential cell detachment. The combined cell population was washed twice with cold PBS. The cells were then resuspended in staining buffer (#PF00018; Proteintech Biotech Co., Ltd., Wuhan, China). Fluorescently labeled anti-caspase-1 antibody (#MA5-32137; 1:1,000; Thermo Fisher Scientific, Waltham, MA, USA) was added, and the cells were incubated for 30 min at 4°C in the dark. After incubation, the cells were washed again with staining buffer to remove unbound antibody. Propidium iodide (PI) solution (#40,710ES03; Yeason Biotech Co., Ltd., Shanghai, China) was then added to the cell suspension, and the cells were incubated for an additional 10 min at room temperature in the dark. Subsequently, samples were analyzed using a flow cytometer (Beckman Coulter CytoFLEX, Miami, FL, USA). FlowJo software was used for data analysis. Cells co-expressing both caspase-1 and PI were defined as pyroptotic. This gating strategy specifically identifies cells that have undergone inflammasome activation and subsequent membrane rupture. Sample analysis was performed immediately after staining on a flow cytometer, and the proportion of double-positive cells was calculated using FlowJo software to represent the pyroptosis rate at each discrete time point.

### Western blot

HPAEpiCs were lysed using RIPA buffer (#89901; Thermo Fisher) supplemented with protease and phosphatase inhibitors after incubation at 0, 4, 8, 12, and 24 h. The lysates were centrifuged at 12,000 × *g* for 15 min at 4°C, and the supernatants were collected. Protein concentration was determined using a commercial BCA kit (#P0012; Beyotime Biotech Co., Ltd., Shanghai, China). Equal amounts of protein were separated by SDS-PAGE and transferred onto PVDF membranes (#LC2002; Thermo Fisher). The membranes were blocked with 5% non-fat milk in TBST for 1 h at room temperature, then incubated overnight at 4°C with primary antibodies against the target proteins. After washing with Tris-buffered saline with Tween 20 (TBST), the membranes were incubated with HRP-conjugated secondary antibodies for 1 h at room temperature. Protein bands were visualized using an ECL detection system (#34580; Thermo Fisher), and band intensities were quantified using ImageJ software. The used antibodies included NLRP3 (#PA5-88709; 1/1000; Thermo Fisher), cleaved caspase-1 (CL-CASP1; #AF4005; Affinity Biotech Co., Ltd. Changzhou, China), Gasdermin D N-terminal domain (GSDMD-N; #ab215203; 1/1,000; Abcam, Cambridge, MA, USA), GAPDH (#ab9485; 1/2,500; Abcam), METTL14 (#ab309096; 1/1,000; Abcam), and goat-anti-rabbit IgG (#ab6721; 1/5,000; Abcam).

### Cell adhesion and invasion assays

The adhesion and invasion assays of HPAEpiCs were conducted on 24-well cell culture plates (NEST). For each time point (0, 4, 8, 12, and 24 h), 2 × 10⁵ monolayer HPAEpiCs were infected with 2 × 10⁶ *S. pneumoniae* D39 suspensions at 37°C in 5% CO₂. In the adhesion assay, after infection, the cells were gently rinsed five times with sterile PBS to remove nonadherent *S. pneumoniae*. Then, HPAEpiCs were digested with 200 μL of 0.25% trypsin-0.02% EDTA for 5 min at 37°C, lysed with 0.025% Triton X-100, serially diluted, and plated onto tryptic soy agar plates with 5% (vol/vol) sheep blood. The plates were incubated at 37°C overnight, and the adherent bacterial colonies were counted. For the invasion assay, following infection and washing to remove nonadherent bacteria as above, cells were incubated with 1 mL fresh DMEM containing 100 μg/mL gentamicin and 10 μg/mL penicillin G for 1 h at 37°C to eliminate extracellular *S. pneumoniae*, then washed five times with sterile PBS. Subsequently, cells were digested, lysed, diluted, plated, and colony-counted as in the adhesion assay. All experiments were repeated three times.

### M^6^A dot blot assay

Total RNA was extracted from control and *S. pneumoniae* D39*-*treated HPAEpiCs using a commercial RNA extraction kit (#R0006; Beyotime). Equal amounts of RNA samples were denatured at 65°C for 5 min and immediately chilled on ice. The denatured RNA (600 ng) was spotted onto a positively charged nylon membrane using a Bio-Dot microfiltration apparatus (Bio-Rad, Hercules, CA, USA). The membrane was cross-linked with UV light and blocked with 5% non-fat milk in TBST for 1 h at room temperature. Following blocking, the membrane was incubated with an anti-m⁶A antibody (#ab284130; 1:5,000; Abcam, Cambridge, MA, USA) overnight at 4°C, followed by washing and incubation with an HRP-conjugated secondary antibody (#ab6721; 1:1,000; Abcam) for 1 h at room temperature. Finally, m⁶A signals were detected using an ECL detection system (#34580; Thermo Fisher) and quantified using ImageJ software.

### Quantitative reverse transcription-polymerase chain reaction

Total RNA was extracted from control and *S. pneumoniae* D39*-*treated HPAEpiCs. The purity and concentration of the extracted RNA were assessed using a spectrophotometer (NanoDrop, Thermo Fisher Scientific, Waltham, MA, USA). Subsequently, 1 μg of total RNA was reverse-transcribed into cDNA using a reverse transcription kit (#11,150ES; Yeason), with the reaction system and conditions configured according to the manufacturer’s protocol. For quantitative reverse transcription-polymerase chain reaction (qRT-PCR), the cDNA was used as a template. Specific primers for the target genes and the reference gene GAPDH were designed and synthesized. qRT-PCRs were performed in a real-time PCR system using SYBR Green PCR Master Mix (#AG11701; Accurate Biology Co., Ltd., Changsha, China). The reaction program consisted of an initial denaturation step, followed by multiple cycles of denaturation, annealing, and extension. Relative expression levels of the target genes were calculated using the 2^⁻ΔΔCt^ method, with GAPDH as the internal reference. Each sample was analyzed in triplicate. The primer sequences are listed in Table 1.

**TABLE 1:** Primer sequences used in qRT-PCR

Gene	Forward (5′−3′)	Reverse (5′−3′)
METTL3	TTGTCTCCAACCTTCCGTAGT	CCAGATCAGAGAGGTGGTGTAG
METTL14 (human)	AGTGCCGACAGCATTGGTG	GGAGCAGAGGTATCATAGGAAGC
METTL14 (mouse)	GAGCTGAGAGTGCGGATAGC	GCAGATGTATCATAGGAAGCCC
WTAP	CTTCCCAAGAAGGTTCGATTGA	TCAGACTCTCTTAGGCCAGTTAC
RBM15	ACGACCCGCAACAATGAAG	GGAAGTCGAGTCCTCACCAC
FTO	ACTTGGCTCCCTTATCTGACC	TGTGCAGTGTGAGAAAGGCTT
ALKBH5	CGGCGAAGGCTACACTTACG	CCACCAGCTTTTGGATCACCA
NLRP3	GATCTTCGCTGCGATCAACAG	CGTGCATTATCTGAACCCCAC
NEK7	CCTTACGACCGGATATGGG	CACTAAATTGTCCGCGACCAA
ASC	TTGGACCTCACCGACAAGCT	CGGTGCTGGTCTATAAAGTGCAG
CASP1	TTTCCGCAAGGTTCGATTTTCA	GGCATCTGCGCTCTACCATC
GSDMD	GTGTGTCAACCTGTCTATCAAGG	CATGGCATCGTAGAAGTGGAAG
IGF2BP1	GCGGCCAGTTCTTGGTCAA	TTGGGCACCGAATGTTCAATC
GAPDH (human)	TGTGGGCATCAATGGATTTGG	ACACCATGTATTCCGGGTCAAT
GAPDH (mouse)	AATGGATTTGGACGCATTGGT	TTTGCACTGGTACGTGTTGAT

### RNA immunoprecipitation assay

To investigate binding interactions between METTL14, IGF2BP1/2/3, and NEK7 in HPAEpiCs, an RNA immunoprecipitation (RIP) assay was performed. HPAEpiCs were lysed in RIP lysis buffer (Geneseed Biotech Co., Ltd., Guangzhou, China) containing RNase-free protease inhibitors. Cell lysates were incubated with magnetic beads conjugated with antibodies against METTL14 (#ab309096; 1/30; Abcam), IGF2BP1 (#ab184305; 1/30; Abcam), IGF2BP2 (#ab117809; 1/30; Abcam), IGF2BP3 (#ab177477; 1/50; Abcam), and NEK7 (#ab133514; 1/50; Abcam), or with IgG as a negative control, respectively. After incubation, the beads were washed extensively, and RNA-protein complexes were eluted. Following washing steps, RNA extraction was performed, and the level of NEK7 RNA was quantified using qRT-PCR to detect RNA enriched in the immunoprecipitated complexes.

### Prediction of m^6^A sites of NEK7

The sequence-based RNA adenosine methylation site predictor database (http://www.cuilab.cn/sramp) was used to predict m^6^A sites of NEK7.

### Dual-luciferase reporter assay

Wild-type and mutant sequences containing the targeted sites (positions 1673, 1722, 1782, and 2868) were cloned into a luciferase reporter vector. HPAEpiCs were co-transfected with the constructed luciferase reporter vectors and either shNC (negative control) or shMETTL14/shIGF2BP1 plasmids. After 48 h, the cells were harvested. Luciferase activity was measured using a luciferase assay kit (#E1500; Promega, Madison, WI, USA), and relative luciferase activity was calculated by normalizing to an internal control using the dual-luciferase reporter assay system (Promega). Each transfection was performed in triplicate to ensure reproducibility.

### RNA stability assessment

RNA stability assessment was performed to verify the stability of NEK7 after METTL14 or IGF2BP1 deficiency in HPAEpiCs. HPAEpiCs were treated with actinomycin D (#53,600ES08; Yeason), then existing NEK7 expression at different time points (0, 4, 8, and 12 h) was analyzed by qRT-PCR.

### Animal study

All animal experimental protocols were approved by the Ethics Committee of MDKN Biotechnology Co., Ltd. (approval number: MDKN-2025-054). Thirty healthy male BALB/c mice (3-week-old, 12–15 g) were procured from Vital River Laboratory Animal Technology Co., Ltd. (Beijing, China). They were housed in a continuously ventilated room at 25°C under a 12-h light/dark cycle with free access to water and food. After 1 week of adaptation, all mice were randomly divided into six groups (*n* = 5): normal, *S. pneumoniae*, *S. pneumoniae* + Lv-shNC, *S. pneumoniae*+Lv-shMETTL14, *S. pneumoniae* + Lv-shM14 + Lv-NC, and *S. pneumoniae* + Lv-shM14 + Lv-NEK7. As per a prior study (17), a mouse pneumonia model was induced by *S. pneumoniae* (D39). *S. pneumoniae* was inoculated on THY sheep blood agar (Aiyan Biotech Co., Ltd, Shanghai, China) overnight and cultured at 37°C with 5% CO_2_ for 14 h, then centrifuged, collected, and resuspended in PBS to 10^9^ CFU/mL. Anesthetized with isoflurane, mice in the *S. pneumoniae* group received 100 μL of PBS containing 1 × 10^8^ CFU (50 μL/nostril) via a 29-gage needle to establish the model, while control mice were injected with an equal inactivated *S. pneumoniae* induced by heat. Two days before modeling, Lv-shMETTL14, Lv-shNC, Lv-NC, or Lv-NEK7 were intravenously injected (viral titer: 1 × 10^9^ TU/mL, 3 μL in 100 μL PBS). After mouse fixation and tail disinfection with 75% ethanol, injection was done at the tail’s 1/3 site at a 30° angle. The needle was retained for 15 min post-injection. The survival rates of mice post-infection were recorded. Afterward, surviving mice were euthanized with 4% isoflurane, and lung tissues were collected for further study.

### Bacterial load measurement

To determine the bacterial load in mouse lungs, the lung tissues were collected at 24 and 48 h post*-S. pneumoniae* infection. The tissues were homogenized in sterile PBS. The homogenates were then serially diluted and plated onto tryptic soy agar plates supplemented with 5% sheep blood. After incubating the plates at 37°C overnight, the colony-forming units (CFU) were counted to quantify the number of viable *S. pneumoniae* in the lung tissues.

### Histological detection

Lung injury was assessed by hematoxylin-eosin (HE) staining. Lung tissues harvested from mice were fixed in a 4% paraformaldehyde solution for 24 h to preserve their structure. Post-fixation, these tissues were embedded in paraffin. Subsequently, the paraffin-embedded specimens were cut into 4-μm-thick sections, which were then stained with HE (#C0105S; Beyotime). Every step of the staining process adhered strictly to the manufacturer’s provided guidelines. Once stained, the sections were examined under a microscope (Olympus, Tokyo, Japan) to assess the extent of lung injury. Lung tissues were scored based on the following indicators: the presence and degree of inflammation, which included inflammatory cell infiltration, hemorrhage, alveolar wall thickening, and alveolar atrophy according to a previous study (18).

For the detection of NEK7, the paraffin-embedded tissue sections underwent an overnight incubation at 4°C with anti-NEK7 antibody (#PA5-101861; 1:200; Thermo Fisher Scientific). This was followed by a 30-min incubation at room temperature with the secondary antibody (goat anti-rabbit; #ab6721; 1:1,000; Abcam). After the antibody incubations, the sections were stained with diaminobenzidine solution (#36,201ES03; Yeason) for 3 min at room temperature. After thorough washing with running water to remove excess stain and then sealed, the stained sections were visualized and imaged using a microscope (Olympus, Tokyo, Japan).

### Statistical analysis

The SPSS 21.0 software (IBM Corp., Armonk, NY, USA) was used for preliminary data analysis. All experiments were conducted using biological replicate samples (independent cell cultures or animal samples). Each biological replicate sample was analyzed three times in technical replicates, and the results were statistically analyzed using the average values. The data were presented in the form of the average values ± standard deviations of at least three independent biological replicate samples. Student’s t-test was employed for comparisons between two groups, while one-way analysis of variance (ANOVA) was applied for comparisons among multiple groups. All statistical analyses, including *post hoc* tests where applicable, were performed using GraphPad Prism software (version 8.0.1; GraphPad Software Inc., San Diego, CA, USA). Mouse survival rates were analyzed using Kaplan–Meier plots, and data were analyzed by the log-rank test. A *P* value < 0.05 was considered statistically significant.

## RESULTS

### Pneumoniae D39-treated HPAEpiCs showed increased pyroptosis and adhesive/invasive capabilities

**Fig 1:**
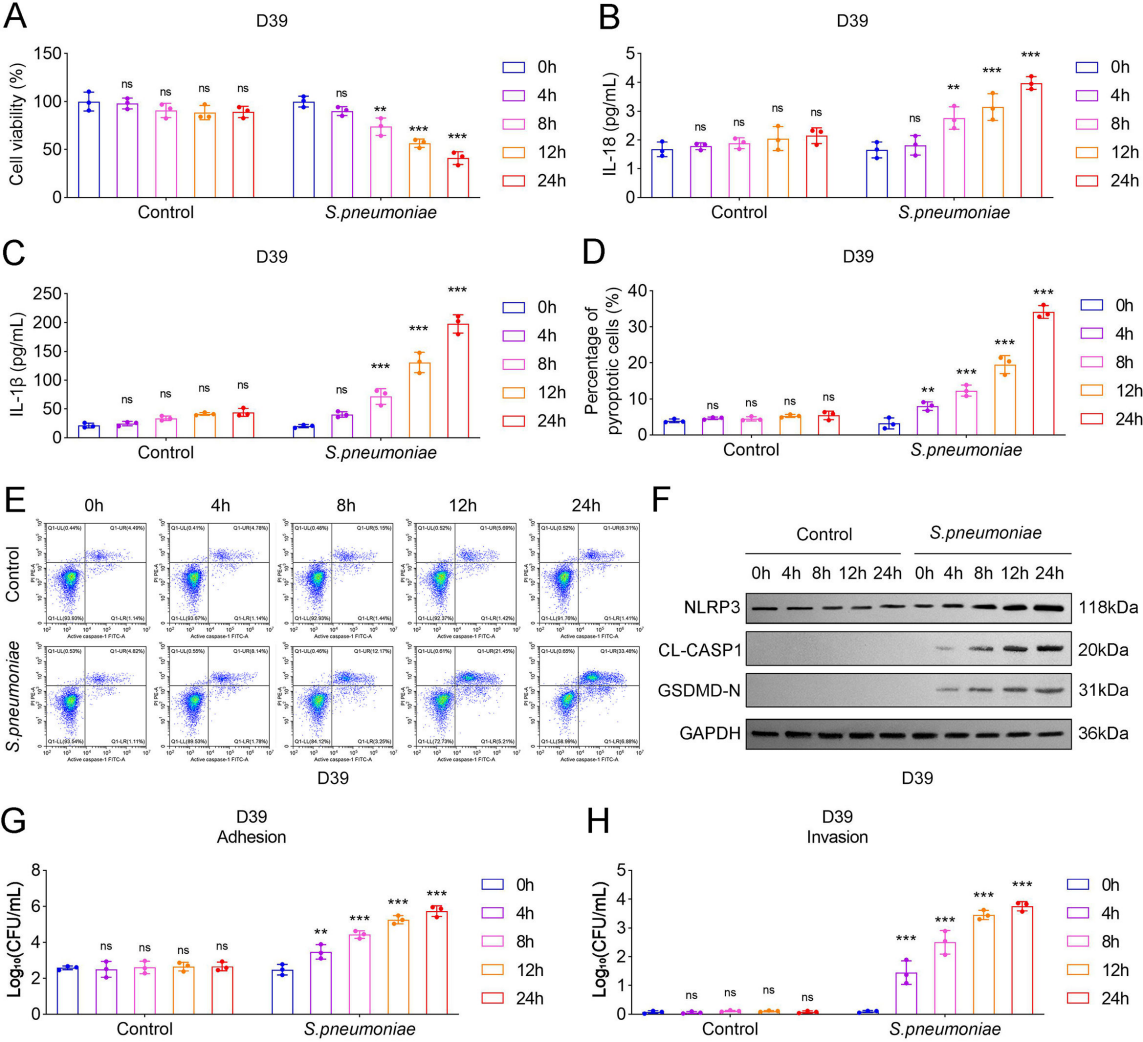
*S. pneumoniae* D39-treated HPAEpiCs showed increased pyroptosis and adhesive/invasive capabilities. (**A**) CCK-8 assay was utilized to assess the survival of HPAEpiCs at 0, 4, 8, 12, and 24 h in each group; ELISA was employed to investigate the inflammatory response in HPAEpiCs through detecting (**B**) IL-18 and (**C**) IL-1β levels. (**D**) Quantification of percentage of pyroptotic cells in HPAEpiCs at 0, 4, 8, 12, and 24 h. (**E**) Flow cytometry was used to show the percentage of cells positive for PI in HPAEpiCs at 0, 4, 8, 12, and 24 h after treatment with *S. pneumoniae.* (**F**) A Western blot was performed to detect the pyroptosis-related protein levels. The (**G**) adhesion and (**H**) invasion assays of *S. pneumoniae* D39 strain to HPAEpiCs cells (*N* = 3; ^**^P < 0.01, ^***^P < 0.001). Data shown are representative of three independent biological replicates.

In this study, HPAEpiCs were infected or left uninfected with *S. pneumoniae* D39. The CCK-8 assay revealed a progressive decline in cell viability in infected cultures over time, with significant reductions observed at 8, 12, and 24 h post-infection. In contrast, cell viability in the control group remained unchanged across all time points (Fig. 1A). To identify the form of cell death involved in the progression of pneumonia induced by *S. pneumoniae* infection, we treated *S. pneumoniae*-infected HPAEpiCs with a panel of cell death inhibitors. Results demonstrated that, compared with the untreated group, treatment with Z-VAD-FMK or VX-765 significantly increased cell viability. In contrast, treatment with Ferrostatin-1, 3-MA, or Necrostatin-1 had no effect on cell viability (Fig. S1). Based on these observations, we focused subsequent investigations on the role of pyroptosis in *S. pneumoniae*-induced pneumonia. ELISA results showed a time-dependent increase in IL-18 and IL-1β concentrations following *S. pneumoniae* infection, with significant elevations at 8, 12, and 24 h, indicating a temporal inflammatory response. Furthermore, IL-18 and IL-1β levels in the control group remained stable at all time points (Fig. 1B and C). Flow cytometry analysis revealed a time-dependent increase in the percentage of pyroptotic cells, with significant elevations observed at 4, 8, 12, and 24 h post-infection. In contrast, the percentage of pyroptotic cells in the control group remained unchanged across all time points (Fig. 1D and E). Additionally, Western blot analysis revealed elevated protein levels of NLRP3, CL-CASP1, and GSDMD-N in *S. pneumoniae* D39-infected HPAEpiCs over time, with significant increases at 4, 8, 12, and 24 h, suggesting activation of the inflammasome complex. However, NLRP3, CL-CASP1, and GSDMD-N levels in the control group remained unaltered at all time points (Fig. 1F). These findings demonstrated that *S. pneumoniae* infection promotes pyroptosis in HPAEpiCs. Furthermore, adhesion and invasion assays revealed that, compared with the control group, *S. pneumoniae* D39 exhibited increased adherence to and invasion of HPAEpiCs at 4, 8, 12, and 24 h post-infection, demonstrating a time-dependent enhancement in the bacterium’s adhesive and invasive capabilities during this period (Fig. 1G and H). In the subsequent study, we chose to infect the HPAEpiCs with *S. pneumoniae* for 12 h. Importantly, replicate experiments performed with the *S. pneumoniae* E1 strain yielded consistent results (Fig. S2). Based on these findings, the *S. pneumoniae* D39 strain was selected for subsequent studies.

### METTL14-mediated m^6^A modification was elevated in *S. pneumoniae* D39**-**treated HPAEpiCs

**Fig 2:**
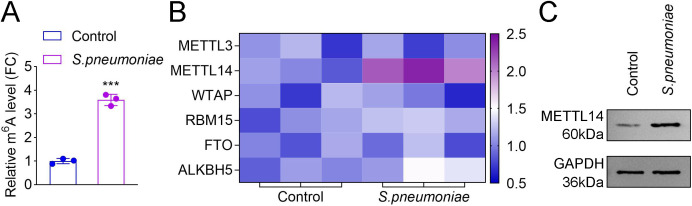
METTL14-mediated m^6^A modification was elevated in *S. pneumoniae* D39-treated HPAEpiCs. (**A**) Dot blot assay was used to analyze the total m^6^A level in the control and the *S. pneumoniae* groups. (**B**) qRT-PCR was used to analyze the expression of METTL3, METTL14, WTAP, RBM15, FTO, and ALKBH5 in the control and the *S. pneumoniae* groups. (**C**) Western blot was performed to detect the protein level of METTL14 in the control and the *S. pneumoniae* groups (*N* = 3; ^***^P < 0.001). Data shown are representative of three independent biological replicates.

In recent years, numerous studies have established a link between m^6^A modification and various pulmonary conditions, including lung injury and lung cancer (12, 19). In this study, our results confirmed that the m^6^A content in *S. pneumoniae* D39-treated HPAEpiCs was significantly higher compared to that in the control group (Fig. 2A). Furthermore, qRT-PCR analysis revealed that METTL14 mRNA expression was significantly upregulated in *S. pneumoniae* D39-treated HPAEpiCs relative to the control group. Additionally, no significant differences were observed in the mRNA levels of METTL3, WTAP, RBM15, FTO, and ALKBH5 between the control and *S. pneumoniae* D39-treated groups (Fig. 2B). Western blot results also demonstrated a marked increase in METTL14 protein levels in *S. pneumoniae* D39-treated HPAEpiCs compared to the control group (Fig. 2C). These findings demonstrated that METTL14-mediated m^6^A modification may play a critical role in *S. pneumoniae* D39*-*infected HPAEpiCs.

### METTL14 inhibition suppressed pyroptosis and adhesive/invasive capabilities in *S. pneumoniae* D39-treated HPAEpiCs

**Fig 3:**
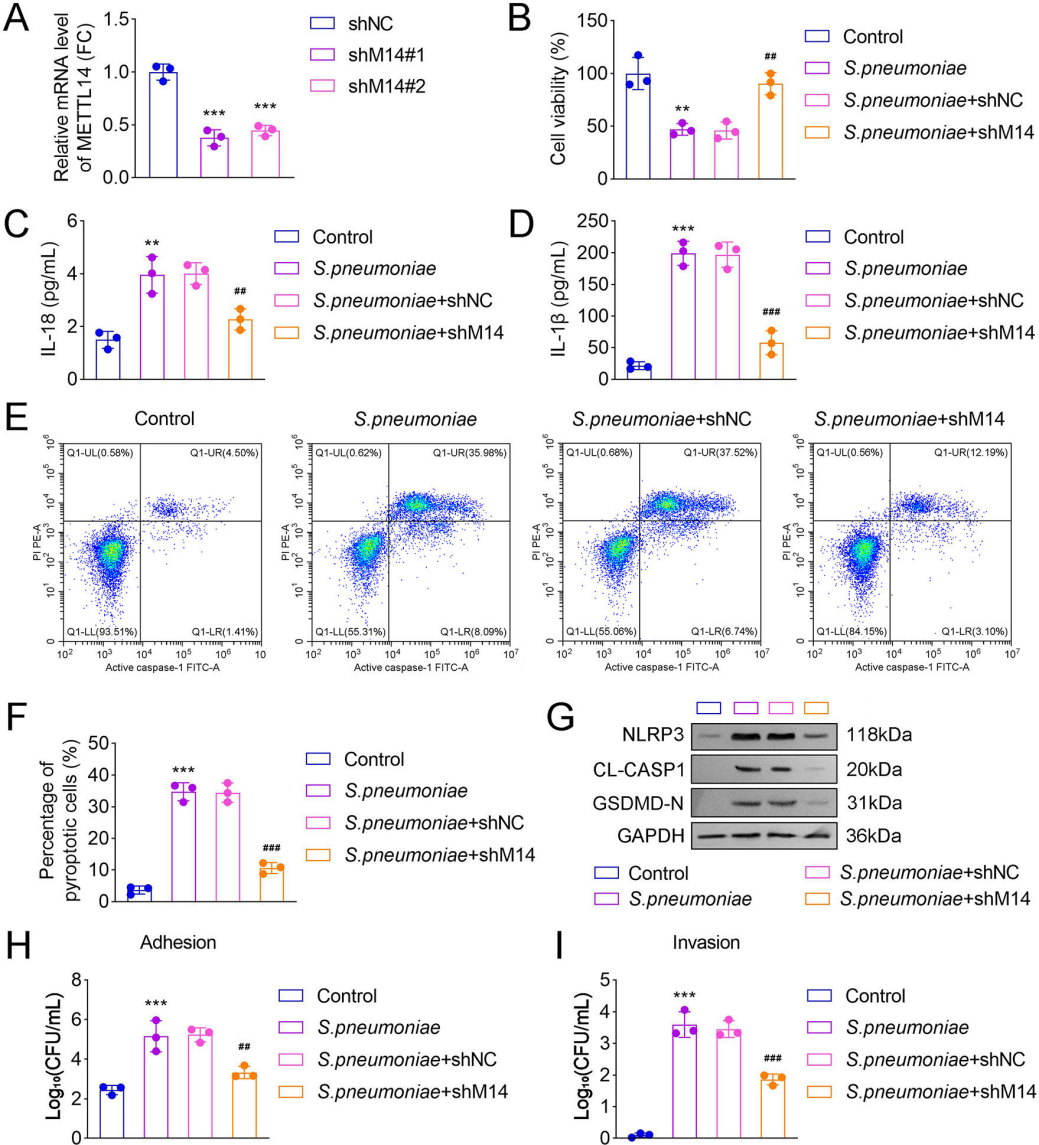
METTL14 inhibition suppressed pyroptosis and adhesive/invasive capabilities in *S. pneumoniae* D39-treated HPAEpiCs. To investigate whether METTL14 knockdown attenuates *S. pneumoniae*-induced pyroptosis and infection capability *in vitro*. HPAEpiCs were transfected with shNC, shMETTL14#1, or shMETTL14#2, followed by *S. pneumoniae* D39 infection. (**A**) Relative mRNA level of METTL14 in HPAEpiCs after transfection of shNC, shMETTL14#1, or shMETTL14#2 was detected by qRT-PCR. (**B**) CCK-8 assay was utilized to assess the cell viability in each group; ELISA was employed to investigate the (**C**) IL-18 and (**D**) IL-1β levels in each group. (**E**) Flow cytometry was used to show the percentage of cells positive for PI in each group. (**F**) Quantification of the percentage of pyroptotic cells in each group. (**G**) Western blot was performed to detect the pyroptosis-related protein levels in each group. The (**H**) adhesion and (**I**) invasion assays of *S. pneumoniae* D39 strain to HPAEpiCs cells (*N* = 3; ^**^P < 0.01, ^***^P < 0.001 vs. the shNC or the control group; ^##^*P* < 0.01, ^###^*P* < 0.001 vs. the *S. pneumoniae* + shNC group). Data shown are representative of three independent biological replicates.

To further explore the role of METTL14 in *S. pneumoniae* D39-induced pyroptosis, shNC and shMETTL14#1/2 vectors were transfected into *S. pneumoniae* D39*-*treated HPAEpiCs. The results demonstrated that METTL14 mRNA levels were significantly decreased following METTL14 inhibition (Fig. 3A). Additionally, cell viability was increased in the shMETTL14 group compared with the *S. pneumoniae* D39 + shNC group (Fig. 3B). Furthermore, the shMETTL14 group exhibited reduced levels of IL-18 and IL-1β, a decreased percentage of pyroptotic cells, and lower protein levels of NLRP3, CL-CASP1, and GSDMD-N relative to the *S. pneumoniae* D39 + shNC group (Fig. 3 through G). These findings indicated that METTL14 suppression reduces pyroptosis in *S. pneumoniae* D39*-*treated HPAEpiCs. In addition, METTL14 inhibition showed decreased *S. pneumoniae* adherence to and invasion of HPAEpiCs (Fig. 3H and I).

### METTL14 inhibition decreased the stability of NEK7 mRNA

**Fig 4:**
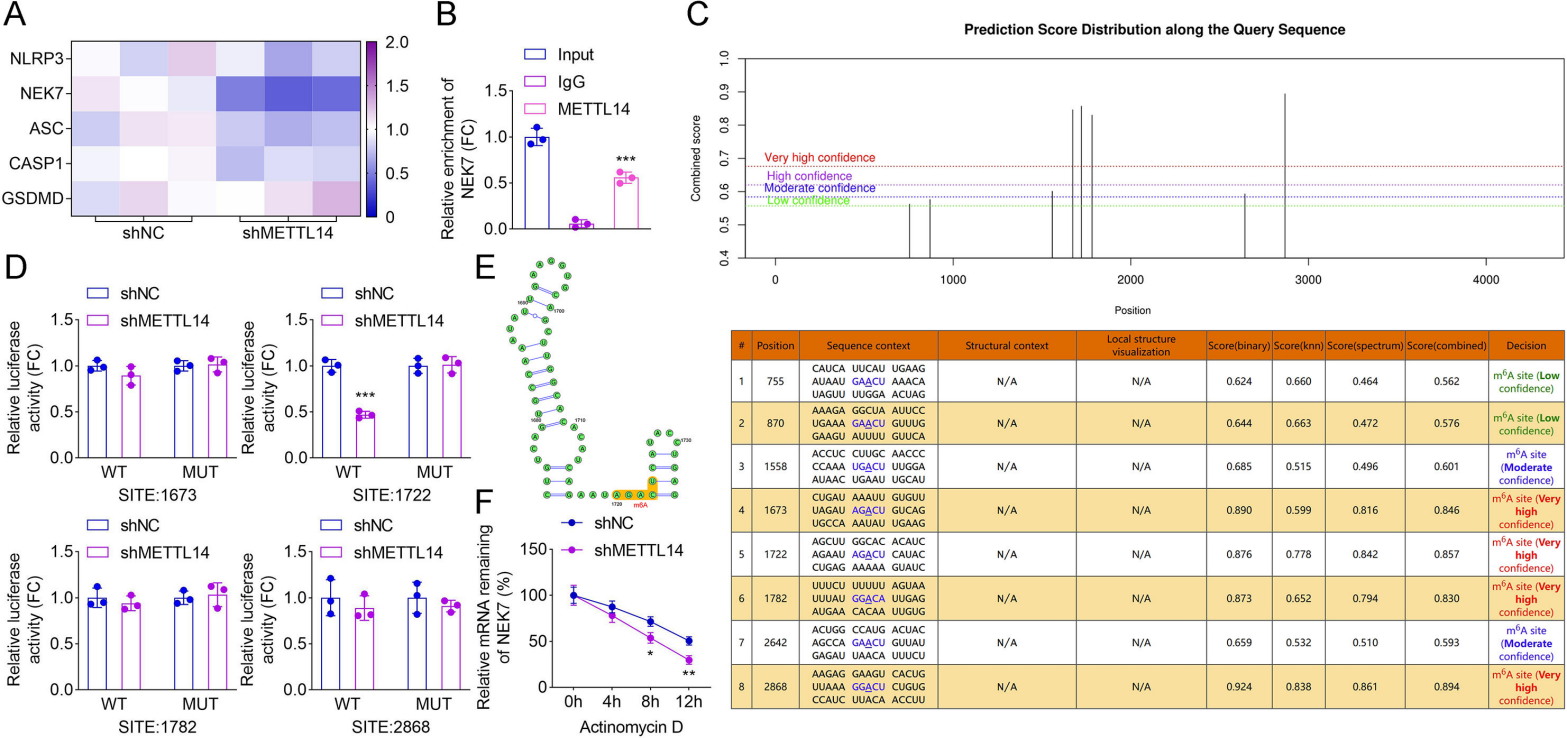
METTL14 inhibition decreased the stability of NEK7 mRNA. (**A**). qRT-PCR was used to analyze the expression of NLRP3, NEK7, ASC, CAS1, and GSDMD in HPAEpiCs after METTL14 inhibition. (**B**) An RIP assay was conducted to examine the interaction between NEK7 and METTL14 in HPAEpiCs. (**C**) Sequence-based RNA adenosine methylation site predictor database was used to predict m^6^A sites of NEK7, and four potential sites (1673, 1722, 1782, and 2868) were predicted. (**D**) Dual-luciferase reporter assay was performed to evaluate the binding of METTL14 and NEK7 at sites 1673, 1722, 1782, and 2868 in HPAEpiCs. (**E**) Predicted secondary structure and m^6^A-related site information of NEK7 gene sequence. (**F**) RNA stability assay was used to detect the existing NEK7 expression when actinomycin D was treated at different time points (0, 4, 8, and 12 h) in HPAEpiCs (*N*=3; ^**^P < 0.01, ^***^P < 0.001). Data shown are representative of three independent biological replicates.

To investigate the downstream mechanisms of METTL14 in pyroptosis regulation, qRT-PCR was performed to evaluate the mRNA expression levels of pyroptosis-related genes, including NLRP3, NEK7, apoptosis-associated speck-like protein containing a CARD (ASC), CASP1, and Gasdermin D (GSDMD), following METTL14 inhibition. The results showed a significant reduction in NEK7 mRNA expression upon METTL14 suppression (Fig. 4A), prompting further investigation into its regulatory role. A RIP assay confirmed the physical interaction between METTL14 and NEK7 mRNA in HPAEpiCs (Fig. 4B). Using an RNA adenosine methylation site predictor database, four potential m^6^A methylation sites on NEK7 mRNA (positions 1673, 1722, 1782, and 2868) were identified (Fig. 4C). Dual-luciferase reporter assays revealed that METTL14 specifically interacted with site 1722 on NEK7 mRNA, compared to sites 1673, 1782, and 2868 (Fig. 4D and E). Additionally, an RNA stability assay demonstrated that METTL14 inhibition accelerated the degradation of NEK7 mRNA in HPAEpiCs, suggesting that METTL14 deficiency reduces NEK7 mRNA stability (Fig. 4F).

### NEK7 overexpression reversed the decreased pyroptosis and adhesive/invasive capabilities induced by METTL14 knockout in *S. pneumoniae* D39-treated HPAEpiCs

**Fig 5:**
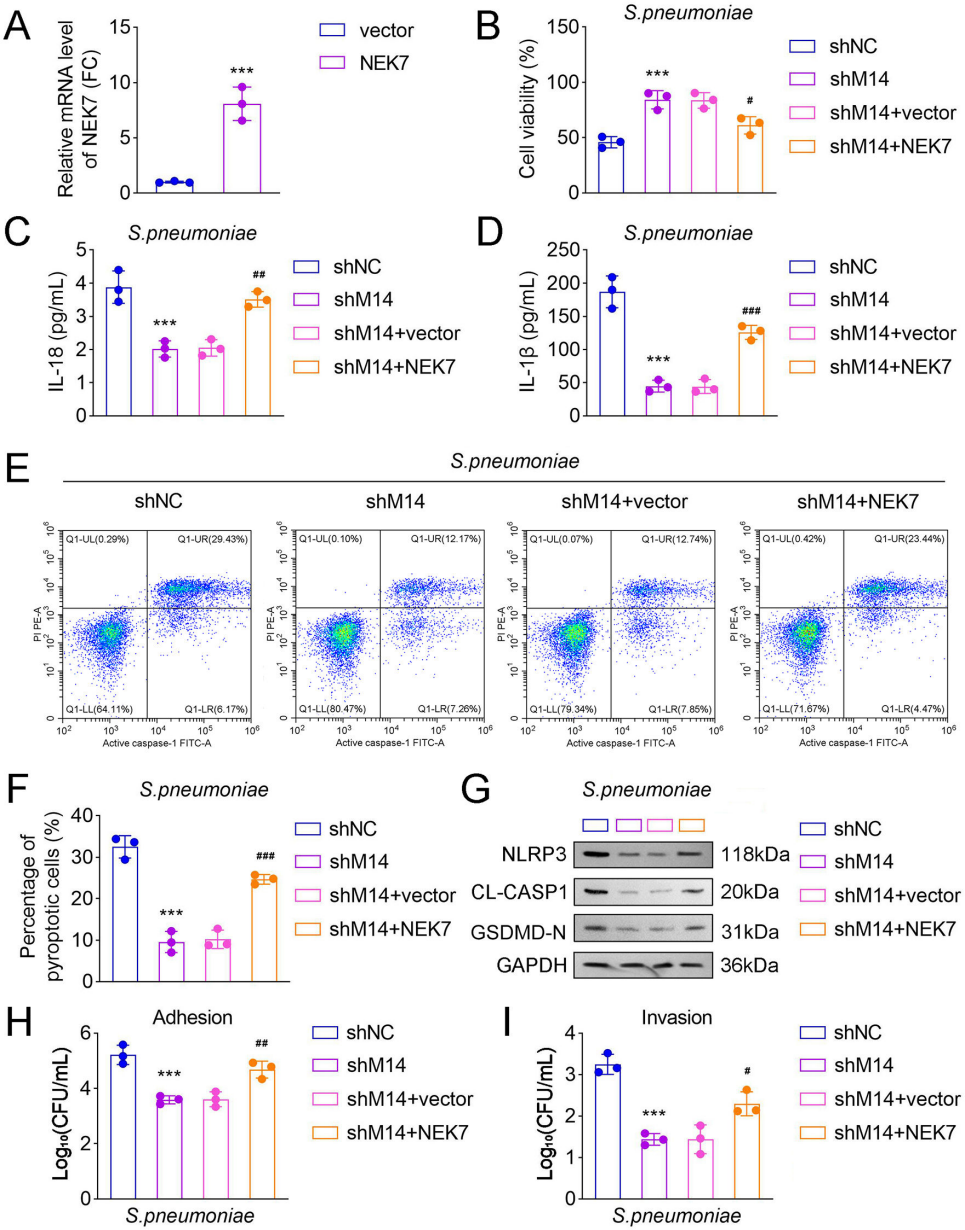
NEK7 overexpression reversed the decreased pyroptosis and adhesive/invasive capabilities induced by METTL14 knockout in *S. pneumoniae* D39-treated HPAEpiCs. (**A**) Relative mRNA level of NEK7 in HPAEpiCs after transfection of NEK7 overexpression vectors was detected by qRT-PCR. (**B**) CCK-8 assay was utilized to assess the cell viability in each group; ELISA was employed to investigate the (**C**) IL-18 and (**D**) IL-1β levels in each group. (**E**) Flow cytometry was used to show the percentage of cells positive for PI in each group. (**F**) Quantification of the percentage of pyroptotic cells in each group. (**G**) Western blot was performed to detect the pyroptosis-related protein levels in each group. The (**H**) adhesion and (**I**) invasion assays of *S. pneumoniae* D39 strain to HPAEpiCs cells (*N*=3; ^***^P < 0.001 vs. the vector or the control group; ^#^*P* < 0.05, ^##^*P* < 0.01, ^###^*P* < 0.001 vs. the shMETTL14 + vector group). Data shown are representative of three independent biological replicates.

To further investigate the functional role of NEK7 in *S. pneumoniae* D39*-*induced pyroptosis, NEK7 overexpression vectors and empty vectors were transfected into *S. pneumoniae* D39*-*treated HPAEpiCs. The results demonstrated that NEK7 mRNA levels were significantly increased following NEK7 overexpression (Fig. 5A). Additionally, cell viability was reduced in the NEK7 overexpression group compared with the shMETTL14+vector group (Fig. 5B). Furthermore, the NEK7 overexpression group exhibited elevated levels of IL-18 and IL-1β, an increased percentage of pyroptotic cells, and higher protein expressions of NLRP3, CL-CASP1, and GSDMD-N relative to the shMETTL14+vector group (Fig. 5C through G). These findings indicated that NEK7 overexpression counteracts the suppression of pyroptosis caused by METTL14 deficiency in *S. pneumoniae* D39*-*treated HPAEpiCs. Moreover, NEK7 overexpression promoted *S. pneumoniae* adherence and invasion in HPAEpiCs (Fig. 5H and I).

### METTL14/IGF2BP1 m^6^A axis mediated NEK7 mRNA stability in HPAEpiCs

**Fig 6:**
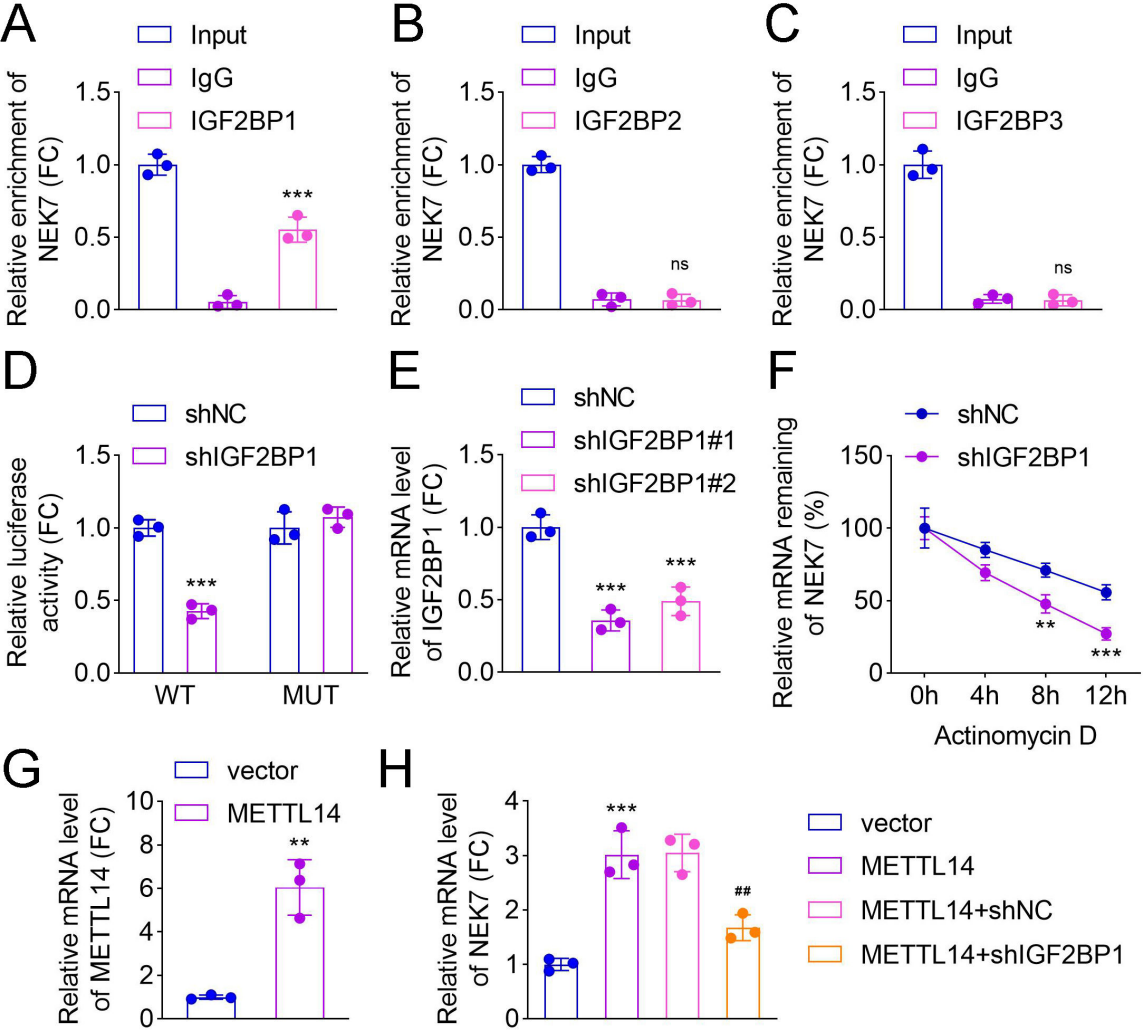
METTL14/IGF2BP1 m^6^A axis mediated NEK7 mRNA stability in HPAEpiCs. RIP assay was conducted to examine the interaction between NEK7 and (**A**) IGF2BP1, (**B**) IGF2BP2, and (**C**) IGF2BP3 in HPAEpiCs. (**D**) Dual-luciferase reporter assay was performed to evaluate the binding of IGF2BP1 and NEK7 in HPAEpiCs. (**E**) Relative mRNA level of IGF2BP1 in HPAEpiCs after transfection of shNC, shIGF2BP1#1, or shIGF2BP1#2 was detected by qRT-PCR. (**F**) RNA stability assay was used to detect the existing NEK7 expression in HPAEpiCs after IGF2BP1 inhibition when actinomycin D was treated at different time points (0, 4, 8, and 12 h); The mRNA levels of (**G**) METTL14 and (**H**) NEK7 in each group were analyzed by qRT-PCR (*N* = 3; ^**^P < 0.01, ^***^P < 0.001 vs. the IgG, shNC, or vector group; ^##^*P* < 0.01 vs. the METTL14 + shNC group). Data shown are representative of three independent biological replicates.

The regulatory function of m^6^A modification depends on its recognition by specific “readers” (20). RIP assays confirmed the interaction between IGF2BP1 and NEK7 mRNA in HPAEpiCs, with no detectable binding observed for IGF2BP2 or IGF2BP3 (Fig. 6A through C). Dual-luciferase reporter assays further validated the specific association between IGF2BP1 and NEK7 in these cells (Fig. 6D). Following transfection of shIGF2BP1#1/2 into HPAEpiCs, a significant reduction in IGF2BP1 expression was observed (Fig. 6E). RNA stability assays revealed that IGF2BP1 knockdown accelerated NEK7 mRNA degradation (Fig. 6F), indicating that IGF2BP1 is critical for maintaining NEK7 mRNA stability in HPAEpiCs. Additionally, qRT-PCR demonstrated that METTL14 overexpression increased both METTL14 and NEK7 mRNA levels (Fig. 6G). Notably, IGF2BP1 silencing significantly reduced NEK7 mRNA levels compared to the METTL14 + shNC group in HPAEpiCs (Fig. 6H).

### NEK7 overexpression reversed the alleviated *S. pneumoniae* D39-induced lung injury induced by METTL14 deficiency *in vivo*

**Fig 7:**
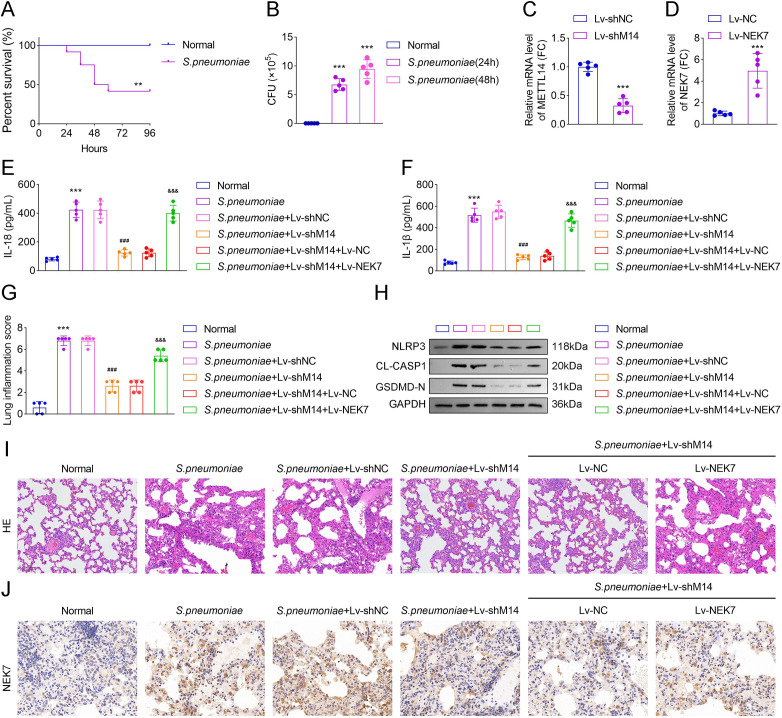
NEK7 overexpression reversed the alleviated *S. pneumoniae* D39-induced lung injury induced by METTL14 deficiency *in vivo*. To determine whether NEK7 mediates METTL14-regulated pyroptosis and lung injury in a mouse pneumonia model. Mice were intranasally infected with *S. pneumoniae* D39 with or without lentiviral-mediated METTL14 knockdown (Lv-shMETTL14) and/or NEK7 overexpression (Lv-NEK7). (**A**) The survival rates of mice post-infection were analyzed by Kaplan–Meier plots. (**B**) CFUs of each group after 24 and 48 h of infection. (**C and D**) Relative mRNA level of METTL14 and NEK7 in the Lv-shMETTL14 and Lv-NEK7 groups was analyzed by qRT-PCR; ELISA was employed to investigate the (**E**) IL-18 and (**F**) IL-1β levels in each group. (**G**) Total lung inflammation score. Rating scale: 0 = absent, 1 = occasionally, 2 = regularly, and 3 = massively; (**H**), Western blot was performed to detect the pyroptosis-related protein levels in each group. (**I**) HE lung tissue staining. (**J**) IHC was performed to analyze the NEK7 expression in the lung tissues (*N* = 5; ^***^P < 0.001 vs. the normal, or the Lv-shNC group; ^##^*P* < 0.01, ^###^*P* < 0.001 vs. the *S. pneumoniae* + Lv shNC group). Data shown are representative of five independent biological replicates.

To evaluate the ability of *S. pneumoniae* to induce lung injury, mice were intranasally administered the bacteria. It was observed that mice infected with *S. pneumoniae* D39 showed decreased survival rates. After infection for approximately 48 h, the survival rate dropped to half (Fig. 7A). Lung damage was assessed via CFU assays, lung inflammation scores, and HE staining. *S. pneumoniae* was detected in mouse lungs 24 and 48 h post-infection (Fig. 7B). In subsequent studies, mice were intranasally administered *S. pneumoniae* for 48 h. Mice in the Lv-shMETTL14 and Lv-NEK7 groups exhibited significantly reduced METTL14 expression and increased NEK7 level compared to the Lv-shNC group (Fig. 7C and D). Furthermore, the *S. pneumoniae* D39*-*infected group demonstrated elevated levels of IL-18 and IL-1β and an increased lung inflammation score compared to the normal group. Notably, METTL14 knockdown significantly decreased lung IL-18 and IL-1β levels and the lung inflammation score relative to the *S. pneumoniae* + Lv-shNC group, and the results were reversed after NEK7 overexpression (Fig. 7E through G). Western blot results showed that METTL14 inhibition decreased the protein levels of NLRP3, CL-CASP1, and GSDMD-N in lung tissues, while the results were reversed after NEK7 overexpression (Fig. 7H). HE staining revealed that *S. pneumoniae* infection induced alveolar septum widening, alveolar fusion, and inflammatory cell infiltration, which were attenuated following METTL14 knockdown. Relative to the *S. pneumoniae* + Lv-shM14+Lv-NC group, NEK7 overexpression aggravated alveolar septum widening, alveolar fusion, and inflammatory cell infiltration (Fig. 7I). Additionally, IHC analysis showed that *S. pneumoniae* infection upregulated NEK7 expression in lung tissues, whereas METTL14 inhibition reduced NEK7 expression compared to the Lv-shNC group. Relative to the *S. pneumoniae* + Lv-shM14 + Lv-NC group, NEK7 overexpression increased NEK7 expression (Fig. 7J).

## DISCUSSION

Our results demonstrated increased pyroptosis in *S. pneumoniae* D39*-*treated HPAEpiCs, evidenced by the time-dependent elevation in IL-18 and IL-1β levels and the upregulation of NLRP3 and CL-CASP1 proteins. In addition, our adhesion/invasion assays further confirm that *S. pneumoniae* D39 actively invades HPAEpiCs, providing a cellular basis for inflammasome sensing. These findings corroborate previous studies highlighting the critical role of pyroptosis in bacterial pneumonia pathogenesis. For instance, Wang et al. demonstrated that *S. pneumoniae* D39*-*infected murine microglia exhibit enhanced reactive oxygen species production, culminating in the secretion of IL-1β and IL-18 and ultimately triggering cellular pyroptosis (21). Additionally, Ma et al. revealed that *S. pneumoniae* infection induces apoptosis and inflammatory injury in alveolar epithelial cells, thereby compromising the alveolar barrier (22). Intriguingly, the contribution of pyroptosis extends beyond bacterial pneumonia to viral and mycoplasma infections. Liu et al. found that in a *Mycoplasma pneumoniae* pneumonia model, Pellino2 promotes inflammation and pyroptosis by facilitating NLRP3 inflammasome ubiquitination and activation (23). Furthermore, Tang et al. and Schifanella et al. identified pyroptosis as a central pathological mechanism in viral pneumonia, including respiratory virus-induced and SARS-CoV-2-associated lung injury (24, 25).

The m^6^A modification has emerged as a crucial post-transcriptional regulatory mechanism in numerous biological processes, including inflammation and cell death (11). In this study, we observed elevated METTL14-mediated m^6^A modification in *S. pneumoniae* D39*-*infected HPAEpiCs. Furthermore, we demonstrated that METTL14 inhibition suppressed pyroptosis in *S. pneumoniae* D39*-*treated HPAEpiCs. Our findings represent one of the first investigations into the role of m^6^A modification in *S. pneumoniae* D39*-*induced pneumonia. Notably, a prior study reported that METTL3 expression is upregulated in *S. pneumoniae* D39*-*infected alveolar epithelial cells, thereby promoting apoptosis and inflammatory injury (22). In other lung diseases, the involvement of m^6^A modification has been extensively documented. Cao et al. revealed that METTL14 contributes to pyroptosis in acute lung injury by stabilizing NLRP3 expression in an IGF2BP2-dependent manner (26). Additionally, Xu et al. demonstrated that global m^6^A modification levels are upregulated in silicosis lung tissues following suppression of the demethylase FTO after 7- and 28-day silica exposure (27).

Our study reveals a novel mechanism by which METTL14 promotes pyroptosis through the regulation of NEK7 mRNA stability. NEK7 has been implicated in the activation of the NLRP3 inflammasome, ultimately leading to pyroptosis. This suggests that NEK7 functions as a critical downstream effector of METTL14 in regulating pyroptosis. Similarly, Wang et al. found that the formation of the NEK7-NLRP3 complex is significantly increased in primary mouse macrophages during *S. pneumoniae* infection, triggering pyroptosis and IL-1β secretion (28). Additionally, prior research demonstrates that the NLRP3 inhibitor Mcc950 reduces IL-1β and IL-18 production, inhibits neutrophil infiltration, and suppresses cell apoptosis by blocking the interaction between NLRP3 and NEK7 (29). Intriguingly, NEK7 has also been shown to contribute to disease progression in other pathologies through m^6^A modification. For instance, Zhou et al. revealed that METTL3 inhibition mitigates NLRP3 inflammasome activation by enhancing ubiquitination of NEK7 in periodontitis (30). Furthermore, Hong et al. demonstrated that METTL3 knockdown inhibits hemin-induced pyroptosis and suppresses m^6^A methylation of NEK7, thereby reducing NEK7 mRNA stability in intracerebral hemorrhage (31). Notably, our study also highlights the role of IGF2BP1 as an m^6^A “reader” that binds to NEK7 mRNA and stabilizes it, a finding that expands our understanding of m^6^A-mediated regulatory mechanisms involving NEK7. Similarly, Huang et al. showed that downregulation of METTL14 alleviates postmenopausal osteoporosis via IGF2BP1-dependent post-transcriptional silencing of SMAD1 (32).

The *in vivo* results corroborate the *in vitro* findings, demonstrating that METTL14 deficiency mitigates *S. pneumoniae* D39*-*induced lung injury. This aligns with prior research indicating that METTL14 contributes to various forms of lung injury, including sepsis- or lipopolysaccharide-induced acute lung injury (26, 33).

In summary, our study identifies METTL14/IGF2BP1-mediated m⁶A modification of NEK7 mRNA as a novel regulatory axis promoting pyroptosis in *S. pneumoniae*-infected alveolar epithelial cells. Unlike direct inhibition of core inflammasome components such as NLRP3 (e.g., MCC950) or caspase-1 (e.g., VX-765)—which, while anti-inflammatory, may compromise host defense and increase susceptibility to bacterial dissemination in clinical settings (34, 35)—targeting the METTL14/IGF2BP1–NEK7 axis offers a more nuanced strategy. By selectively modulating NEK7 stability rather than broadly suppressing inflammasome activation, this approach may attenuate pathological hyperinflammation without abolishing protective immunity. Indeed, complete blockade of IL-1β or NLRP3 signaling has been linked to increased risk of severe human infections (36, 37), underscoring the need for balanced immunomodulation. Thus, the METTL14/IGF2BP1–m⁶A–NEK7 pathway provides both mechanistic insight into pneumonia pathogenesis and a potentially safer therapeutic target for fine-tuning pyroptotic responses during bacterial infection.

However, several limitations warrant further investigation. First, the findings derived from cellular and animal models necessitate validation through clinical studies to confirm translational relevance. Second, the functional significance of the identified m^6^A modification site (1722) on NEK7 mRNA requires detailed characterization to clarify its physiological role. Third, we did not include genetically modified non-virulent *S. pneumoniae* strains (e.g., capsule-deficient Δcps mutants or autolysin-deficient ΔlytA mutants) to further exclude the potential interference of specific bacterial virulence factors or proinflammatory fragments from bacterial lysis in triggering pyroptosis. These limitations will be further explored in our future investigation.

## Data Availability

The data sets used and/or analyzed during the current study are available from the corresponding author on reasonable request.

## SUPPLEMENTAL MATERIAL

Supplemental figures iai.00474-25-s0001.docx Fig. S1 and S2.

ASM does not own the copyrights to Supplemental Material that may be linked to, or accessed through, an article. The authors have granted ASM a non-exclusive, world-wide license to publish the Supplemental Material files. Please contact the corresponding author directly for reuse.
