# Supplementary material for: METTL14/IGF2BP1 m6A axis promotes pyroptosis in Streptococcus pneumoniae-induced pneumonia by regulating NEK7 mRNA stability
Source: Infect Immun. 2026 Feb 12;94(3):e00474-25. doi: 10.1128/iai.00474-25 (PMC12974142; doi:10.1128/iai.00474-25)
Supplement: Supplemental figures — Fig. S1 and S2. [file iai.00474-25-s0001.docx]

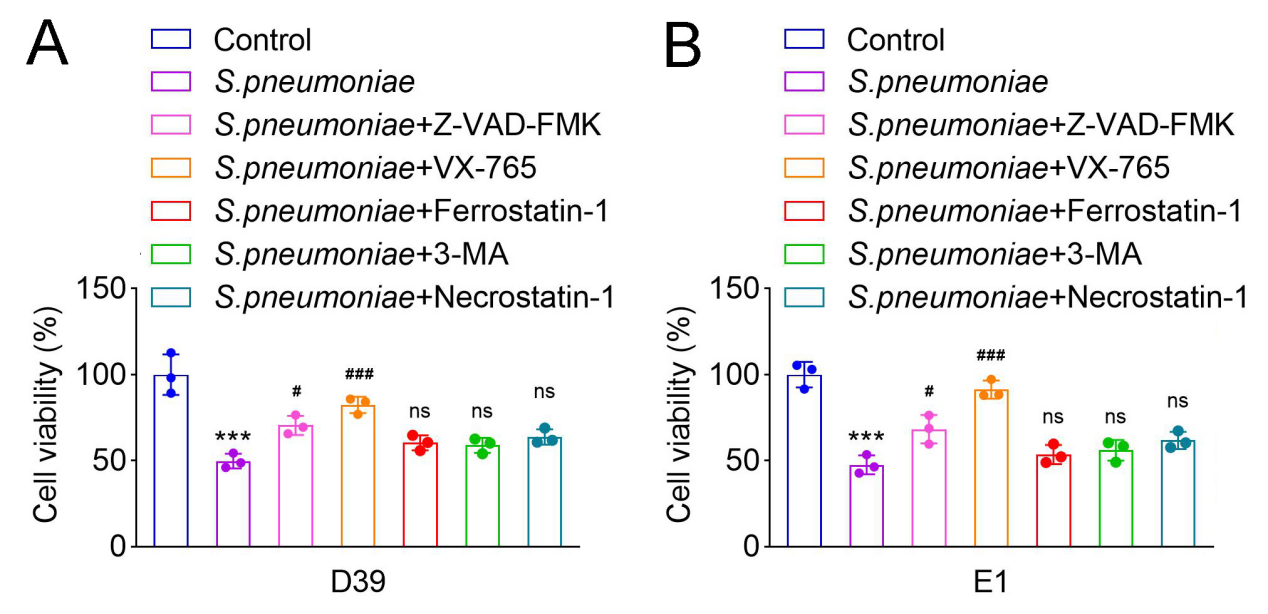


**Supplemental Figure 1.** Z-VAD-FMK or VX-765 treatment enhances cell viability, while ferroptosis, autophagy, and necroptosis inhibition show no effect.

**A**, D39 cells and **B**, E1 cells were infected with *S. pneumoniae* or co-treated with Z-VAD-FMK, VX-765, Ferrostatin-1, 3-MA, or Necrostatin-1. Cell viability was assessed by CCK-8 assay and expressed as a percentage relative to the control group. (N=3; ^***^*p* < 0.001 vs. the control group; ^#^*p* < 0.05, ^###^*p* < 0.001 vs. the *S. pneumoniae* group) Data shown are representative of three independent biological replicates.


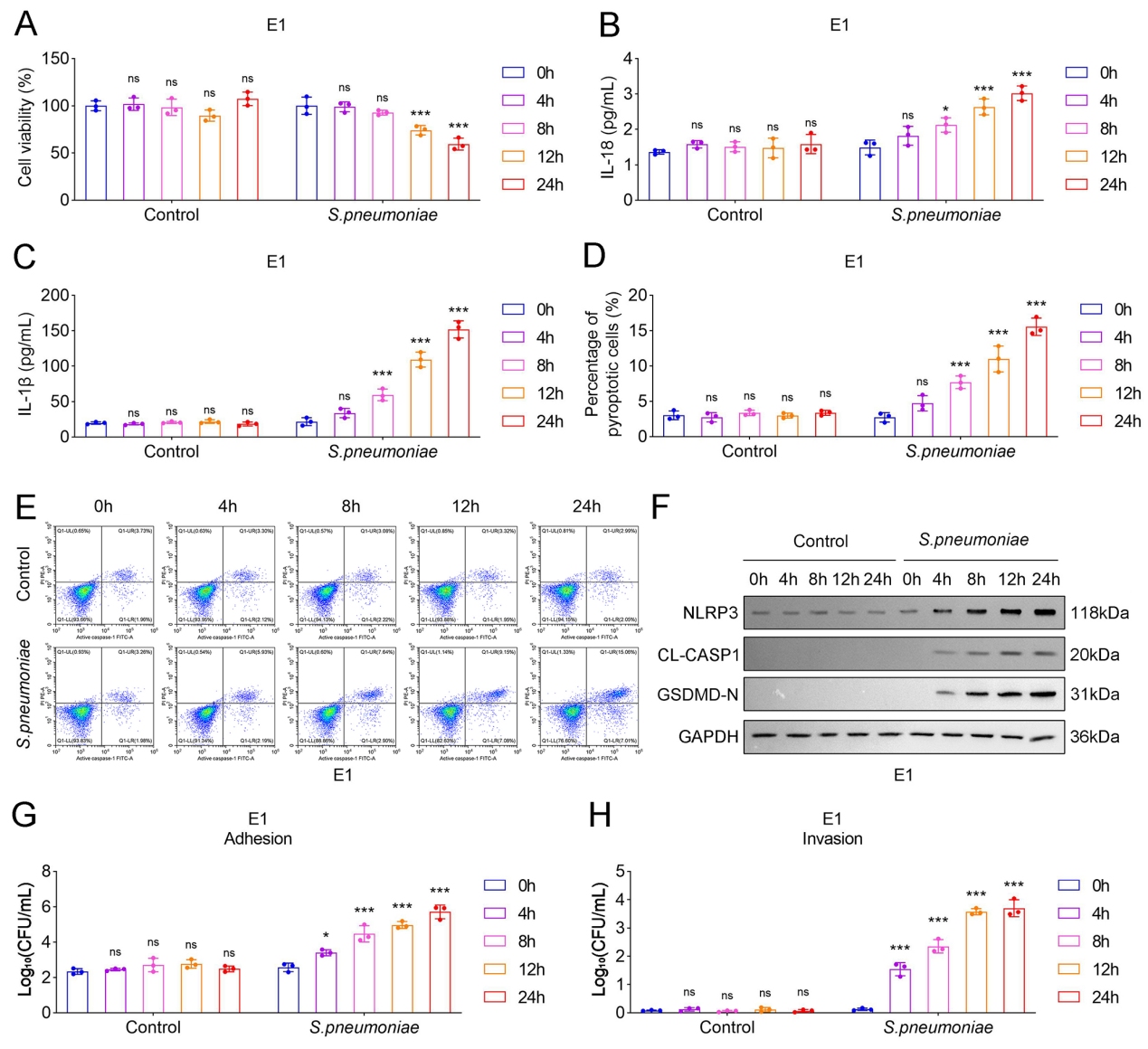


**Supplemental Figure 2.** *S. pneumoniae* E1-treated HPAEpiCs showed increased pyroptosis and adhesive/invasive capabilities.

**A**, CCK-8 assay was utilized to assess the survival of HPAEpiCs at 0, 4, 8, 12, and 24 h in each group; ELISA was employed to investigate the inflammatory response in HPAEpiCs through detecting **B,** IL-18 and **C**, IL-1β levels; **D**, Quantification of percentage of pyroptotic cells in HPAEpiCs at 0, 4, 8, 12, and 24 h; **E**, Flow cytometry was used to show the percentage of cells positive for PI in HPAEpiCs at 0, 4, 8, 12, and 24 h after treatment with *S. pneumoniae*; **F**, Western blot was performed to detect the pyroptosis-related protein levels; The **G**, adhesion and **H**, invasion assays of *S. pneumoniae* E1 strain to HPAEpiCs cells. (N=3; ^*^*p* < 0.05, ^***^*p* < 0.001) Data shown are representative of three independent biological replicates.
